# Adipokine resistin predicts anti-inflammatory effect of glucocorticoids in asthma

**DOI:** 10.1186/1476-9255-8-12

**Published:** 2011-05-26

**Authors:** Sirpa Leivo-Korpela, Lauri Lehtimäki, Katriina Vuolteenaho, Riina Nieminen, Hannu Kankaanranta, Seppo Saarelainen, Eeva Moilanen

**Affiliations:** 1 Department of Respiratory Medicine, Tampere University Hospital, Tampere, Finland; 2 The Immunopharmacology Research Group, University of Tampere School of Medicine and Tampere University Hospital, Tampere, Finland; 3 Department of Respiratory Medicine, Seinäjoki Central Hospital, Seinäjoki, Finland

## Abstract

**Background:**

Adipokines are protein mediators secreted by adipose tissue. Recently, adipokines have also been involved in the regulation of inflammation and allergic responses, and suggested to affect the risk of asthma especially in obese female patients. We assessed if adipokines predict responsiveness to glucocorticoids and if plasma adipokine levels are associated with lung function or inflammatory activity also in non-obese (body mass index (BMI) ≤ 30 kg/m^2^) women with newly-diagnosed steroid-naïve asthma.

**Methods:**

Lung function, exhaled NO, plasma levels of adipokines leptin, resistin, adiponectin and adipsin, and inflammatory markers were measured in 35 steroid-naïve female asthmatics and in healthy controls. The measurements were repeated in a subgroup of asthmatics after 8 weeks of treatment with inhaled fluticasone. Adipokine concentrations in plasma were adjusted for BMI.

**Results:**

High baseline resistin concentrations were associated with a more pronounced decrease in serum levels of eosinophil cationic protein (ECP) (r = -0.745, p = 0.013), eosinophil protein X (EPX) (r = -0.733, p = 0.016) and myeloperoxidase (MPO) (r = -0.721, p = 0.019) during fluticasone treatment. In asthmatics, leptin correlated positively with asthma symptom score and negatively with lung function. However, no significant differences in plasma adipokine levels between non-obese asthmatics and healthy controls were found. The effects of resistin were also investigated in human macrophages in cell culture. Interestingly, resistin increased the production of proinflammatory factors IL-6 and TNF-α and that was inhibited by fluticasone.

**Conclusions:**

High resistin levels predicted favourable anti-inflammatory effect of inhaled glucocorticoids suggesting that resistin may be a marker of steroid-sensitive phenotype in asthma. High leptin levels were associated with a more severe disease suggesting that the link between leptin and asthma is not restricted to obesity.

## Background

Asthma is a chronic inflammatory airway disease characterised by cough, chest tightness and wheezing, and it is associated with reversible or variable airway obstruction. However, the diagnosis and follow-up of the disease are currently based on symptoms and lung function measurements rather than on assessing the underlying inflammatory process [1]. Several asthmatic phenotypes with different inflammatory mechanisms have been described suggesting that asthma is not a single disease entity but a syndrome with different underlying causes and mechanisms [2]. The efficacy of treatment with inhaled glucocorticoids seems to vary between asthmatic phenotypes, and phenotype-specific predictors of treatment response are needed.

Adipokines like leptin, adiponectin, resistin and adipsin are protein mediators secreted by adipocytes and macrophages within the adipose tissue [3]. Leptin and resistin are usually pro-inflammatory, while adiponectin has mainly anti-inflammatory properties [3]. Leptin levels increase in obesity [4] and leptin has therefore been suggested to belong to the factors explaining the relation between obesity and asthma. Some studies suggest that leptin affects asthma also independently of body mass index (BMI) [5,6]. Adiponectin has been demonstrated to have anti-inflammatory properties [3,7] and it is associated with lower risk for asthma in women regardless of BMI [8]. There are only a few publications on resistin in human asthma with conflicting results [9-11]. Larochelle et al [9] found higher resistin levels in asthmatics and the levels were increased with disease severity, while Kim et al [10] suggested that resistin may have a protective effect against asthma. The role of adipsin in asthmatic inflammation has not been studied previously. There is limited data on adipokines in non-obese asthmatics and only a little information how treatment with inhaled glucocorticoids influence the circulating levels of adipokines.

As discussed above, there are some evidence suggesting connections between adipokines and asthma. However, further studies are needed to understand the role of adipokines in the pathogenesis of, and more importantly, in predicting treatment responses in different phenotypes of asthma. Nuclear factor κB (NF-κB) is a transcription factor inducing the expression of many pro-inflammatory genes. Inhaled glucocorticoids exert their anti-inflammatory effects through a wide variety of mechanisms, of which inhibition of NF-κB is one of the most important [12]. Interestingly, also adipokine resistin has been linked to NF-kB at two levels; its expression is enhanced by inflammatory factors IL-1, IL-6, TNF-α and LPS [13] which all are known activators of NF-κB. In addition, pro-inflammatory effects of resistin are partly mediated through activation of the NF-κB pathway [14]. Therefore resistin may have a role as a factor or a predictor in steroid-responsive asthma.

The aim of the present study was to assess if plasma levels of resistin or other adipokines would predict the responsiveness to inhaled corticosteroids, and if adipokines are associated with lung function, symptoms or inflammatory activity in newly diagnosed asthma in non-obese (BMI ≤ 30 kg/m^2^) female subjects. We found that high baseline resistin levels predicted favourable response to inhaled fluticasone, while high leptin levels were associated with poor lung function and more symptoms.

## Methods

### Subjects

Thirty-five steroid-naive, non-smoking female asthmatics (mean age 34 yrs, range 20-57 yrs) with BMI ≤ 30 kg/m^2 ^(range 18-30 kg/m^2^) were recruited. The diagnosis of asthma was based on symptoms and reversible or variable airway obstruction (β_2_-agonist induced increase in FVC or FEV_1 _≥ 12% and 200 ml, or diurnal variability in PEF ≥20%, or exercise induced decrease in FEV_1 _≥ 15%). Thirty-two age- and sex-matched non-smoking healthy controls with similar BMI, no asthmatic symptoms and normal lung function served as controls. Both groups were free from any other chronic diseases.

### Study protocol

Lung function, asthma symptom score, plasma levels of adipokines, serum levels of other inflammatory markers, and exhaled nitric oxide (NO) were measured in asthmatics and in controls. The asthmatics also filled in an asthma symptom questionnaire. The same measurements were repeated in 11 asthmatics after 8 weeks of treatment with inhaled fluticasone propionate (Flixotide Diskus, GSK, Ware, UK, 500 μg b.i.d. during weeks 1-4, and 250 μg b.i.d. during weeks 5-8). The study was approved by the ethics committee of Tampere University Hospital and all subjects gave their written informed consent.

### Adipokines and inflammatory markers

Venous blood was collected for the assessment of plasma levels of adipokines (resistin, leptin, adiponectin, adipsin), serum levels of immunoglobulin E (IgE), eosinophil cationic protein (ECP), eosinophil protein X (EPX), myeloperoxidase (MPO), interleukin 6 (IL-6), and blood eosinophil count (EOS). Adipokines were determined by enzyme-immuno-assay (EIA) by using commercial reagents (DuoSet ELISA, R&D Systems Europe Ltd, Abindgon, U.K). As plasma adipokine levels are dependent on the amount of adipose tissue, adipokine levels were adjusted for BMI by dividing the measured concentration by BMI. Radioimmunoassay (ECP RIA, EPX RIA and MPO RIA, Pharmacia AB, Uppsala, Sweden) was used to measure ECP, EPX and MPO levels. Immunoluminometry was used to measure IgE, and IL-6 was measured by EIA (PeliPair ELISA, Sanquin, Amsterdam, Netherlands). The detection limits and inter-assay coefficients of variation, respectively, were 15,6 ng/l and 4.0% for resistin, 15.6 ng/l and 3.9% for leptin, 15.6 ng/l and 2.0% for adiponectin, 4.0 ng/l and 3.8% for adipsin, 2.0 μg/l and 4.2% for ECP, 3.0 μg/l and 5.4% for EPX, 8.0 μg/l and 6.2% for MPO and 0.6 ng/l and 6.1% for IL-6.

### Exhaled NO and lung function

Exhaled NO was measured with a Sievers NOA 280^® ^NO-analyzer (Sievers Instruments, Boulder, CO, USA) at exhalation flow rates of 100, 175 and 370 ml/s with a mouth pressure of 9 cmH_2_O. The analyzer was calibrated daily with a known NO concentration (103 parts per million (ppm), AGA, Sweden) and before every subject with filtered NO-free air. Bronchial NO flux and alveolar NO concentration were calculated for each subject using the method described by Tsoukias and George [15,16]. Airway function was measured with Vmax 20 C spirometer (Sensor-Medics, Yorda Linda, CA, USA) before and after 400 μg of inhaled salbutamol.

### Asthma symptoms questionnaire

Asthma symptoms were recorded by using written symptom questionnaire. Cough, chest tightness, wheezing and nocturnal asthma symptoms were each scored from 0 to 3 yielding a total score from 0 to 12 points [17].

### Cell culture

Human THP-1 monocyte/macrophage cell line (American Type Culture Collection, Manassas, VA, USA) was used. The cells were cultured at 37°C in humidified 5% carbon dioxide atmosphere in RPMI 1640 medium adjusted to contain 2 mM L-glutamine, 10 mM HEPES, 1 mM sodium pyruvate, 4.5 g/l glucose, and 1.5 g/l bicarbonate, and supplemented with 10% heat-inactivated fetal bovine serum (all obtained from Lonza Verviers SPRL, Belgium), penicillin (100 units/ml), streptomycin (100 μg/ml) and amphotericin B (250 ng/ml) (all obtained from Invitrogen, Paisley, UK), and 0.05 mM 2-mercaptoethanol. The cells were differentiated to macrophages by adding the phorbol ester 12-O-tetradecanoylphorbol-13-acetate (TPA, 100 nM) for 72 h at the time of seeding of the cells on 24-well plates. Cells were serum starved for 16 h before the experiments were started. Resistin (recombinant human resistin; PeproTech, Inc., Rocky Hill, NJ, USA) and fluticasone (Sigma Chemical Co, St. Louis, MO, USA) were added in fresh culture medium, and the cells were incubated for 24 h. Culture medium was collected and stored at -20°C until assayed. The concentrations of human IL-6 (PeliPair ELISA, Sanquin, Amsterdam, Netherlands) and human TNF-α (R&D Systems, Minneapolis, MN, USA) were determined by ELISA. The detection limits and intra-assay coefficients of variation, were 7.8 ng/l and 4.8% for TNF-α and 0.6 ng/l and 6.0% for IL-6, respectively.

### Statistics

Normality of the distributions of plasma adipokines and other parameters were analysed with Kolmogorov-Smirnov's test. Differences in adipokine levels between asthmatics and controls were analysed with t-test or Mann-Whitney test, where appropriate. Spearman's rho was used to analyse correlations between adipokine levels and lung function indices, other inflammatory markers or symptom scores. Changes in plasma levels of adipokines and other markers of inflammation during fluticasone treatment were analysed with a paired t-test or Wilcoxon's test, where appropriate. A stepwise multiple linear regression analysis was used to determine if the correlations between lung function indices and the levels of plasma adipokines were explained by BMI. Results from the cell culture experiments were analyzed by using one-way ANOVA followed by Dunnett multiple comparisons test. Results are presented as mean ± SEM for normally distributed data and as median [interquartile range] for non-normally distributed data. A p-value < 0.05 was considered as significant. SPSS 15.0.1 software (SPSS Inc., Chicago, Illinois, USA) was used in the statistical analysis.

## Results

Subject characteristics are given in Table 1. There were no differences in age or BMI between asthmatics and controls. Asthmatics had higher serum levels of EPX and IgE, and higher blood eosinophil count and bronchial NO flux than controls.

**Table 1:** Subject characteristics.

	Asthmatics	Controls	p-value
N	35	32	
Age (yrs)	33.9 ± 2.1	33.8 ± 2.1	0.980
BMI (kg/m^2^)	23.1 ± 0.5	22.8 ± 0.5	0.627
FEV_1_(% pred)	90 ± 1.9	96 ± 3.2	0.125
ECP (μg/l)	11.2 [6.9 - 19.9]	9.2 [6.1 - 14.4]	0.105
EPX (μg/l)	29.6 [20.8 - 61.1]	18.3 [16.3 - 27.4]	0.003
MPO (μg/l)	218.6 [138.0 - 325.0]	246.8 [155.7 - 317.4]	0.716
EOS (10^9^/l)	0.23 [0.16 - 0.44]	0.15 [0.10 - 0.20]	<0.001
IgE (IU/l)	87.0 [25.0 - 204.0]	24.5 [11.0 - 41.0]	0.002
IL-6 (ng/l)	3.8 [2.6 - 5.3]	3.0 [2.3 - 5.0]	0.327
J_Br,NO _(nl/s)	2.6 ± 0.3	0.7 ± 0.1	<0.001
C_Alv _(ppb)	1.2 ± 0.3	1.1 ± 0.1	0.671

BMI, body mass index

FEV_1_, forced expiratory volume in 1 second

ECP, eosinophil cationic protein

EPX, eosinophil protein X

MPO, myeloperoxidase

EOS, blood eosinophil count

IgE, immunoglobulin E

IL-6, interleukin 6

J_Br,NO_, Bronchial NO flux

C_Alv_, Alveolar NO concentration

Values are presented as mean ± SEM for normally distributed data and as median [interquartile range] for non-normally distributed data.

Leptin and resitin levels were normally distributed, while distribution of adiponectin and adipsin were non-normal. As plasma adipokine levels are dependent on the amount of adipose tissue, adipokine levels were adjusted for BMI by dividing the measured concentration by BMI. There were no significant differences in BMI-adjusted plasma adipokine levels between asthmatics and healthy controls (Table 2).

**Table 2:** Plasma levels of adipokines in asthmatics and controls.

	Asthmatics	Controls	p-value
N	35	32	
Resistin (ng/l)/BMI (m^2^/kg)	0.5 [0.4 - 0.8]	0.5 [0.5 - 0.7]	0.603
Leptin (ng/l)/BMI (m^2^/kg)	0.5 [0.5 - 1.1]	0.6 [0.4 - 0.8]	0.366
Adiponectin (ng/l)/BMI (m^2^/kg)	165 ± 9.5	176 ± 13	0.490
Adipsin (ng/l)/BMI (m^2^/kg)	32 ± 1.3	33 ± 1.3	0.813

Adipokine values were adjusted for BMI (body mass index)

Values are presented as mean ± SEM for normally distributed data and as median [interquartile range] for non-normally distributed data.

### Predicting treatment responses

**Figure 1:**
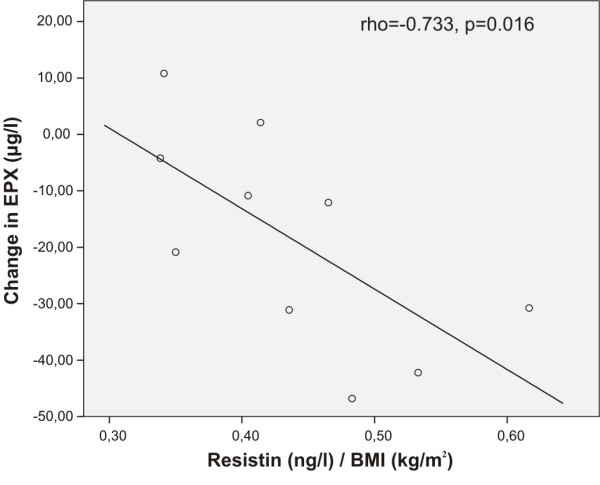
**Correlation between baseline resistin and fluticasone-induced change in EPX**. Baseline BMI-adjusted resistin correlated negatively with the change in serum levels of eosinophil protein X (EPX) during inhaled fluticasone treatment (Spearman's rank correlation), i.e. the higher the baseline resistin the larger the decrease in EPX levels in response to inhaled fluticasone.

**Figure 2:**
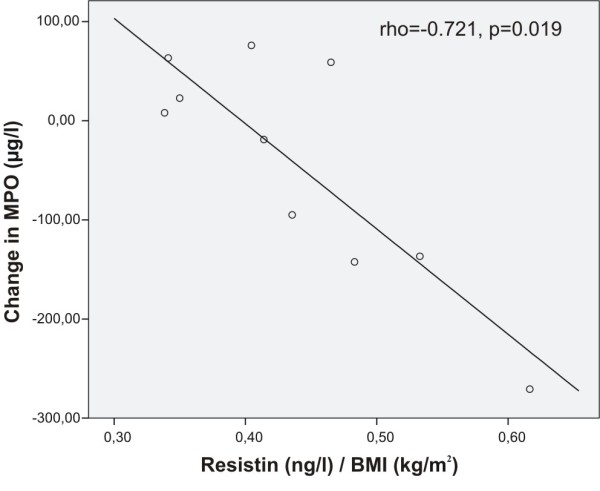
**Correlation between baseline resistin and fluticasone-induced change in MPO**. Baseline BMI-adjusted resistin correlated negatively with the change in serum levels of myeloperoxidase (MPO) during inhaled fluticasone treatment (Spearman's rank correlation), i.e. the higher the baseline resistin the larger the decrease in MPO levels in response to inhaled fluticasone.

Interestingly, pre-treatment resistin levels seemed to predict the anti-inflammatory effect of inhaled fluticasone. Baseline BMI adjusted resistin correlated negatively with change in serum levels of ECP (rho = -0.745, p = 0.013), EPX (rho = -0.733, p = 0.016, Figure 1), and MPO (rho = -0.721, p = 0.019, Figure 2) during fluticasone treatment, i.e. the higher the pre-treatment resistin the better the response to inhaled fluticasone. The other adipokines did not correlate significantly with fluticasone-induced changes in the inflammatory markers.

Treatment with inhaled fluticasone decreased plasma adipsin levels but had no effects on other adipokines. Fluticasone treatment decreased also serum levels of ECP and EPX, reduced bronchial NO flux and asthma symptoms, and improved lung function (Table 3).

**Table 3:** Plasma adipokines and other parameters before and after 8-week treatment with fluticasone in 11 asthmatics.

	Before treatment	After treatment	p-value
Resistin (ng/l)/BMI (m^2^/kg)	0.4 [0.3 - 0.5]	0.4 [0.4-0.5]	0.722
Leptin (ng/l)/BMI (m^2^/kg)	0.5 [0.4 - 1.1]	0.7 [0.2-1.0]	0.722
Adiponectin (ng/l)/BMI (m^2^/kg)	154.4 ± 20.1	146.4 ± 21.0	0.271
Adipsin (ng/l)/BMI (m^2^/kg)	27.5 ± 1.5	24.9 ± 1.8	0.026
ECP (μg/l)	16.0 [8.5 - 46.8]	12.4 [6.2 - 21.4]	0.026
EPX (μg/l)	47.2 [28.8 - 68.4]	22.3 [16.6 - 45.1]	0.013
MPO (μg/l)	218.6 [163.5 - 409.1]	199.7 [144.7 - 266.8]	0.534
FEV_1_(% pred)	85 ± 4.0	95 ± 5.5	0.032
J_Br,NO _(nl/s)	3.6 ± 0.4	0.6 ± 0.1	<0.001
C_Alv _(ppb)	1.5 ± 0.6	1.3 ± 0.1	0.705
Symptom score	6.0 [4.0 - 10.0]	0 [0.0 - 0.0]	0.005

ECP, eosinophil cationic protein

EPX, eosinophil protein X

MPO, myeloperoxidase

FEV_1_, forced expiratory volume in 1 second

J_Br,NO_, Bronchial NO flux

C_Alv_, Alveolar NO concentration

Adipokine values were adjusted for BMI (body mass index).

Values are presented as mean ± SEM for normally distributed data and as median [interquartile range] for non-normally distributed data.

### Correlations between adipokines and other parameters

**Figure 3:**
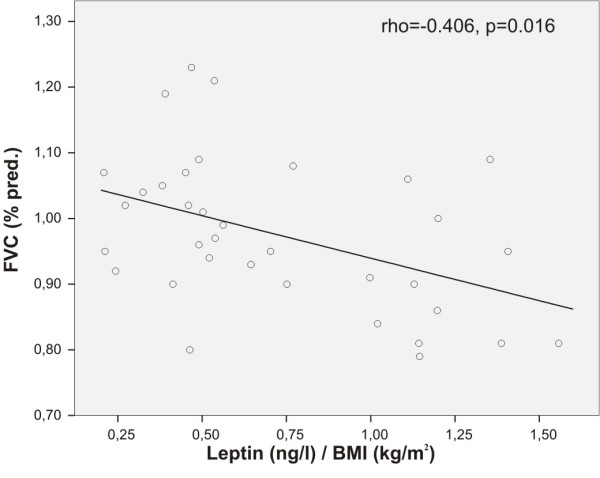
**Correlation between leptin and FVC in steroid-naïve asthmatics**. BMI-adjusted plasma concentrations of leptin correlated negatively with forced vital capacity (FVC, % predicted) in asthmatics (Spearman's rank correlation), i.e. the higher the BMI adjusted leptin level the lower the FVC (% predicted).

**Figure 4:**
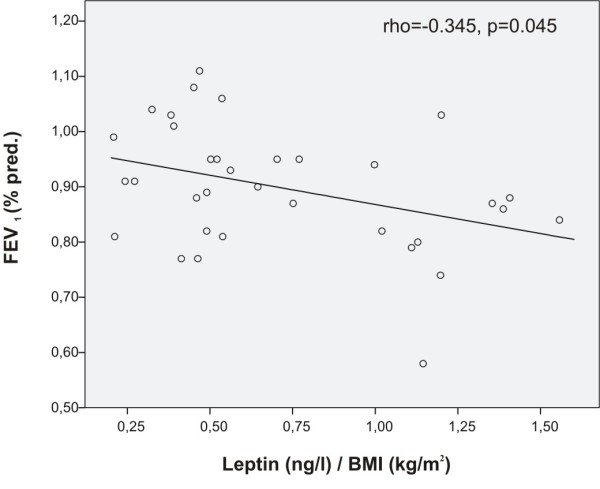
**Correlation between leptin and FEV**_**1 **_**in steroid-naïve asthmatics**. BMI-adjusted plasma concentrations of leptin correlated negatively with forced expiratory volume in 1 second (FEV_1_, % predicted) in asthmatics (Spearman's rank correlation), i.e. the higher the BMI adjusted leptin level the lower the FEV_1 _(% predicted).

In asthmatics, BMI adjusted leptin correlated positively with asthma symptom score (rho = 0.371, p = 0.031) and negatively with lung volumes VC% predicted (rho = -0.445, p = 0.007), FVC% predicted (rho = -0.406, p = 0.016, Figure 3) and with FEV_1_% predicted (rho = -0.345, p = 0.045, Figure 4), i.e. the higher the leptin level, the poorer the lung function and the more symptoms. In contrast, BMI adjusted resistin correlated positively with lung volumes VC % predicted (rho = 0.383, p = 0.023) and FVC % predicted (rho = 0.439, p = 0.008) in asthmatics. Adiponectin and adipsin had no correlations with indices of lung function, symptoms or serum markers of inflammation.

As both lung function and plasma adipokines are related to BMI, we tested if the above mentioned correlations between adipokines and lung function are explained by BMI. We conducted a stepwise multiple linear regression analysis with lung function as the dependent variable, and BMI and adipokine levels as independent variables. Correlation of BMI adjusted resistin with VC % predicted and FVC % predicted were explained by changes in BMI. However, BMI adjusted leptin was an independent predictor of VC % predicted, FVC % predicted and FEV_1 _% predicted.

### The effects of resistin on IL-6 and TNFα production in human macrophages

**Figure 5:**
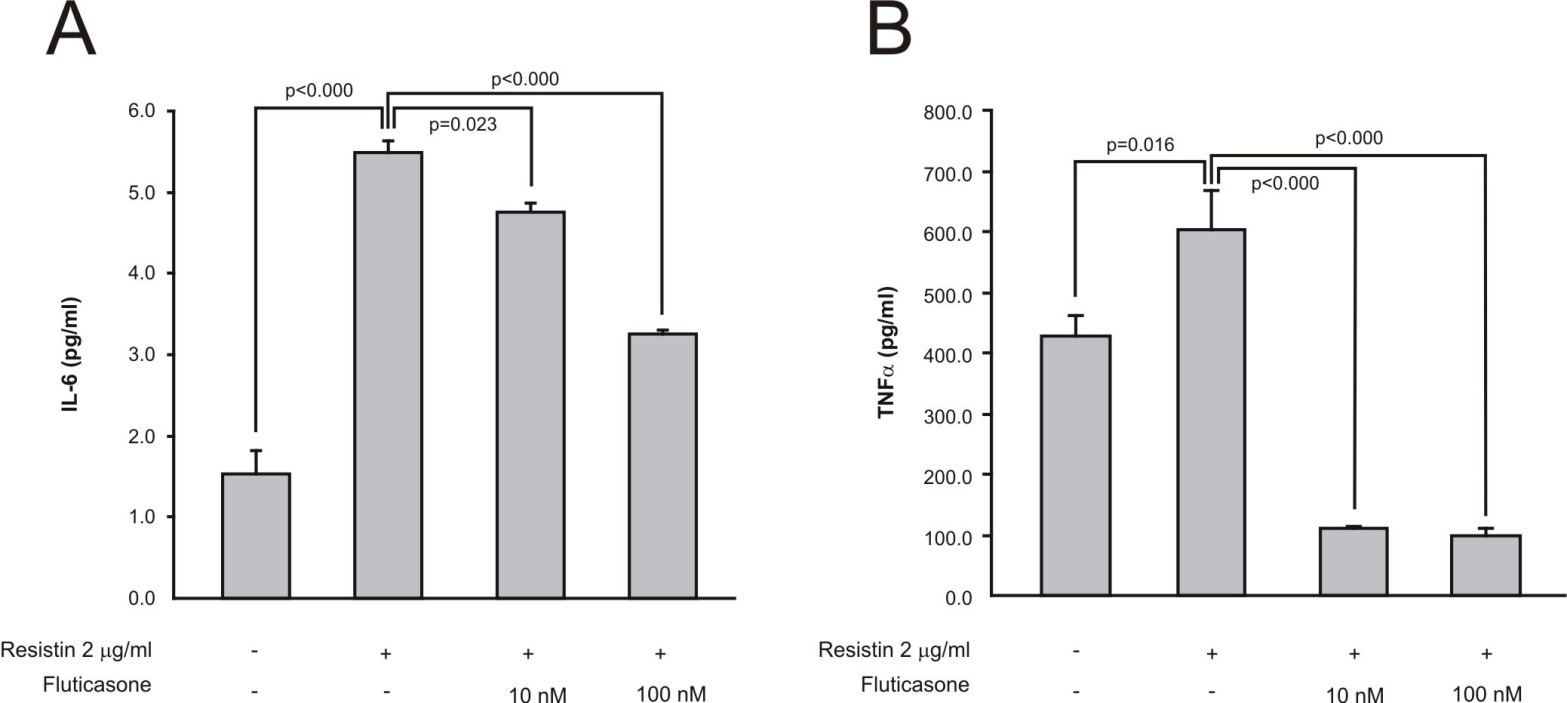
**Resistin enhanced cytokine production in human macrophages, and that was reversed by fluticasone**. Human THP-1 macrophages were cultured for 24 h with resistin (2 μg/ml) in the absence and in the presence of fluticasone (10 - 100 nM). Thereafter interleukin-6 (IL-6, A) and tumor necrosis factor alpha (TNFα, B) concentrations were measured in the culture media by ELISA. Results are expressed as mean ± SEM.

Because resistin levels were associated with favourable anti-inflammatory activity of fluticasone, we studied the effects of this adipokine on human THP-1 macrophages. Interestingly, resistin (0.1 - 2 μg/ml) increased production of proinflammatory cytokines IL-6 and TNF-α in THP-1 cells in a concentration-dependent manner. Moreover, fluticasone (10 and 100 nM) significantly reduced resistin-induced IL-6 and TNF-α production in (Figure 5).

## Discussion

In the present study, we investigated the role of adipokines in asthma in non-obese steroid-naive female patients. The main finding was that high pre-treatment resistin levels were associated with a more pronounced decrease in serum levels of inflammatory markers during fluticasone treatment indicating a better steroid-response. In addition, high plasma leptin levels were associated with poorer lung function and increased symptoms suggesting that leptin is related to the severity of asthma also in non-obese patients.

Resistin is associated with different inflammatory states [3], but there are only a few previous publications on resistin in patients with asthma. LaRochelle *et al *showed that steroid-treated patients with moderate to severe asthma had higher levels of resistin than controls, and resistin levels were increased with increasing disease severity [9]. On the contrary, Kim and colleagues found that resistin levels were lower in atopic asthmatic children than in healthy controls, and resistin was associated with lower markers of atopy or bronchial responsiveness [10]. However, Arshi *et al *did not find any differences in resistin levels between pediatric patients with asthma and healthy children [11]. In the present study including non-obese women with newly diagnosed steroid-naïve asthma, we found that baseline resistin concentrations correlated with anti-inflammatory effects of inhaled fluticasone suggesting that resistin may be a feature and biomarker of steroid-sensitive phenotype of asthma. This relation may be explained by the finding that resistin is an endogenous agonist of Toll-like receptor 4 (TLR4) which leads to activation of various genes involved in asthmatic inflammation through NF-kB pathway [18]. Accordingly, we found here that resistin was able to enhance the production of proinflammatory cytokines IL-6 and TNF-α in human macrophages and interestingly, this effect was inhibited with fluticasone. Also, the expression of resistin itself has been reported to be enhanced by inflammatory factors like IL-1, IL-6, TNF-α and LPS by an NF-κB dependent manner [13,14]. Therefore high resistin levels may reflect an asthmatic phenotype characterized by increased NF-κB activity and hence favourable response to glucocorticoids, the anti-inflammatory action of which is primarily based on their suppressive effect on NF-κB [12].

We found that in non-obese female asthmatics the levels of adipokines were not different from healthy controls. Previously, conflicting results on the levels of adipokines in patients with asthma have been published. Leptin has been reported to be increased [5,6,19,20] or normal [10,21,22] in asthma, resistin either increased [9] or decreased [10], and adiponectin either decreased [8,23] or normal [10,21,22]. There are no previous publications on adipsin in asthma. The conflicting results are likely explained by differences in patient selection. Asthma is often considered as a single disease entity, but it is actually a syndrome with many different pathological pathways ultimately leading to quite similar clinical presentation: variable airway obstruction with chest tightness, wheezing and cough [2]. The role of adipokines quite likely varies between these different inflammatory processes. In addition, there are patient-related contributing factors like age, sex, fat distribution in the body, menopause, atopy, comorbidities and drugs, but there is insufficient data on the detailed effects, mechanisms and significance of these factors so far.

Interestingly, BMI-adjusted leptin levels were associated with poorer lung function and more symptoms in the present study in non-obese steroid-naïve asthmatics. This is in line with a previous study showing an inverse correlation between leptin levels and lung function in non-obese healthy subjects [24] suggesting that leptin is associated with lung function regardless of BMI. Leptin has been reported to induce the production of pro-inflammatory mediators TNF-α, IL-6 and IL-12 [25]. This may further augment asthmatic inflammation and might explain the association of leptin to asthma severity.

We also found that inhaled glucocorticoids decreased plasma levels of adipsin but had no effect on other adipokines. This may be explained by the previous finding that glucocorticoids down-regulate the expression of adipsin gene [26]. In line with the negative effect of fluticasone on leptin in the present study, Radetti's and Heuck's groups have reported previously that leptin secretion was not affected by inhaled corticosteroids [27,28]. However, there are no previous studies on the effect of inhaled glucocorticoids on the levels of other adipokines than leptin.

## Conclusions

In non-obese women with newly-diagnosed steroid-naïve asthma, high resistin levels predicted favourable anti-inflammatory effect of inhaled glucocorticoids suggesting that resistin may be a feature and biomarker of steroid-sensitive phenotype of asthma. High leptin levels were associated with a more severe asthma suggesting that the link between adipokine leptin and asthma is not restricted to obesity.

## Competing interests

The authors declare that they have no competing interests.

## Authors' contributions

SL-K and LL performed the statistical analysis and drafted the manuscript. KV carried out the cell culture experiments. LL and EM developed the protocol and equipment and supervised the exhaled NO measurements. RN and EM were responsible for the analyses of adipokines and inflammatory markers. HK and SS handled the patient recruitment and clinical treatment. All authors participated in the design of the study, and they all read and approved the final manuscript.
